# Data on the working population in Spain related to training, workplace conditions and accident rates

**DOI:** 10.1016/j.dib.2018.10.175

**Published:** 2018-11-03

**Authors:** M.A. Mariscal, E.M. López-Perea, S. García-Herrero, J.R. López-García, S. Herrera

**Affiliations:** a University of Burgos, Spain; b University of Cantabria, Spain

## Abstract

Obtaining data on worker accident rates is necessary in order to analyze the causes and variables involved in the occurrence of said accidents. The majority of these data, collected after the accident occurs, do not consider the employee׳s working conditions. Here are presented the data on workplace accidents and the conditions of the workers by analyzing the generic data supplied as part of the 7th National Survey of Workplace Conditions (EWCS) in Spain, conducted in 2011. These data will yield the variables needed to determine if the information on workplace risks provided by the survey respondents has an appreciable effect on the occurrence of occupational accidents in the working population, and will also be used to explore other variables.

**Specifications table**

**Table:** 

Subject area	Population health
More specific subject area	Occupational risk prevention – Occupational health
Type of data	Tables, figures and explanatory text
How data were acquired	Spanish Ministry of Labor and Social Security - http://encuestasnacionales.oect.es/
Data format	Tables
Experimental factors	Survey of 8892 workers in different production sectors in relation to their working conditions
Experimental features	Use of different variables contained in the survey to study the relationship with the probability of workplace accidents
Data source location	Spain
Data accessibility	Data are with this article.

**Value of data**

• The number of respondents was high. As a result, an analysis of the survey can be used to draw conclusions for a wide range of workers that reflect their situation in terms of their working conditions
• The dataset shows doctor visits for work-related health problems. This variable is not usually included in other studies and will allow researchers to observe its relationship with workplace accidents.
• The dataset includes the results of the training and information given to workers, which may allow for new studies to be carried out on these variables and their impact on the accident rate.
• The dataset provides information for future health and safety at work studies, with special interest for Health and Safety Technical Experts and medical professionals.

## 1 Data

The data were retrieved from the 7th National Survey of Workplace Conditions, study that is periodically conducted by the National Institute for Workplace Health and Safety to provide updated information on the conditions under which employees work in Spain [1].

The survey data were collected between 19 October 2011 and 21 February 2012.

The respondents were aged 16 and older and represented every economic activity in every part of Spain, with the exception of Ceuta and Melilla.

To collect the data, a total of 8892 workers were interviewed at their residences.

The questionnaire contains 62 questions and is structured into the following thematic areas:

• work status and contract type,
• data on work center,
• type of work,
• physical agents,
• chemical and biological pollutants,
• safety conditions,
• design of the workplace,
• work load and psychosocial factors,
• prevention methods,
• work schedule,
• prevention activities,
• information and training,
• violent conduct in the workplace,
• health problems,
• personal information.

Sample error – for a confidence level of 95.5% (two sigmas) and P = Q, the error for the sample group is ±1.06% [2].

It is important to note that the survey specialists subsequently applied the weighted coefficients shown in Table 1 to the data. These coefficients were intended to adjust the sample, based on the area of activity and the size of the workforce at the company, to the actual distribution of the occupational universe considered in the study and to more accurately reflect the real landscape of workers in Spain [2].

**Table 1:** Weighted coefficients.

	1–10	11–49	50–249	250 or more
Agriculture, livestock, forestry and fishing	0.85705279	0.64512531	0.59527518	0.36505732
Chemical, sanitation and mining	0.91369871	0.94861726	0.63661339	0.62436562
Metal	1.13373986	1.01240032	0.91819092	0.82656415
Manufacturing industry	0.78213572	0.94648195	0.78873261	0.57182255
Construction	1.18422164	1.39239229	0.72668685	0.62392982
Commerce and repairs	1.20567816	1.43139239	0.92279056	0.72015132
Hotels	0.97191677	0.98203491	0.83612237	0.59999526
Transport and storage	1.10275509	1.21849885	0.75389600	0.71646392
Communications, financial, scientific and administrative activities	1.08567185	1.16045881	0.84898609	0.85295099
Public administration and education	1.25880517	1.00897268	0.86627675	0.86878192
Healthcare and veterinary activities and social services	1.14463920	1.10137259	0.81554043	1.05676891
Cultural and sports activities and personal services	1.05701812	1.13254789	0.78459853	0.97477475

The weighting factors were calculated based on data from the annual average of the 2011 Survey of the Working Population (EPA 2011).

As a result, the data for the different variables will be presented both weighted and unweighted to clarify how the final (unweighted) data were obtained. These final data do not match those obtained from the National Workplace Health and Safety Institute through the National Observatory for Working Conditions [3].

The frequency tables that will be presented below, and that will provide a basis for later study, will be considered without this weighting factor, with each worker׳s answers being considered as individually relevant to our study.

Many authors have considered working conditions from a standpoint associated with worker accident rates. The variables selected in this paper are deemed to be suitable for subsequent analysis.

The contract type and accident rate are factors considered in the existing literature [4], [5], [6], [7]. Other aspects, such as the worker׳s experience and the area of activity, are recurring topics for various authors [8], [9], [10]. Lastly, the implementation of a safety culture in the company, based on aspects like the information and training given to employees to make them aware of the risks and safety measures in use in the workplace, has also been considered in previous studies [11], [12].

## 2 Experimental design, materials and methods

In this section, we consider the frequencies and categories associated with the selected variables, namely:

• contract type,
• work experience at the work center,
• information on the risks to the worker at his/her job,
• training received by the worker involving the prevention of risks on the job,
• accidents suffered in the previous two years,
• doctor visits for work-related health problems,
• area of activity of the work center.

### 2.1 Contract type

The data for this variable were obtained from questions Q-02 and Q-03 of the questionnaire.

Question Q-02 determines if the worker is a salaried employee or belongs to another category related to self-employed, a cooperative, etc. (freelancers, other non-salaried category).

For salaried workers, question Q-03 involves the type of contract. Excluded in this question are answers from workers classified as “not salaried” in Q-02. Table 2 shows the frequencies for each category in question Q-02. Table 3 shows the summarized frequencies for the subsequent analysis relating both questions.

**Table 2:** Question Q-02 on the NSWC.

**Occupational status**	No. of answers		Grouped answers	
**Q-02**	Weighted	Unweighted	Weighted	Unweighted
Salaried employee enrolled in Social Security (with contract)	7062	7157	7062	7157
Salaried employee not enrolled in Social Security (no contract)	258	251	1830	1735
Independent freelancer with no salaried employees (business owner w/o employees)	777	728		
Dependent freelancer with no salaried employees	369	347		
Business owner with employees	357	335		
Member of a cooperative	37	42		
Helps in family business	27	27		
Other	5	5		
**Total**	**8892**	**8892**	**8892**	**8892**

**Table 3:** Questions Q-02/Q-03 on the NSWC.

**Occupational status/contract type**	No. of answers		Grouped answers	
**Q-02/Q-03**	Weighted	Unweighted	Weighted	Unweighted
Permanent	5220	5293	7062	7157
Permanent seasonal	412	419		
Per job or service	915	909		
Temporary for production reasons	272	287		
Temporary	152	156		
In training	20	19		
Intern	16	16		
Temporary through an agency	37	40		
Other	18	18		
Not salaried (Q-02)			1830	1735
**Total**	**7062**	**7157**	**8892**	**8892**

### 2.2 Time on the job

These values correspond to the answers to Q-13 on the survey questionnaire. This variable is continuous and discrete, and ranges in value between 1 month and 52 years. The data from the survey are grouped into eight categories, and range from periods of less than 1 month to periods longer than 10 years. The data are shown in Table 4.

**Table 4:** Question Q-13 on the NSWC.

**Time on the job**	No. of answers	
**Q-13 grouped by intervals**	Weighted	Unweighted
1 month	229	233
2–6 months	553	549
7 months to 1 year	526	515
1–3 years	1332	1297
3–6 years	1655	1654
6–10 years	1438	1431
>10 years	3134	3186
DK/NA	26	27
**Total**	**8892**	**8892**

### 2.3 Perception of the information on the risks involved in the job

The workers surveyed were asked to give their opinion on the quality/quantity of the information received in terms of the risk that their current job poses to their health and safety. This aspect is reflected in question 48 of the EWCS, the results of which are shown in Table 5.

**Table 5:** Question Q-48 on the NSWC.

**Information on risks**	No. of answers	
**Q-48**	Weighted	Unweighted
Very well informed	2381	2410
Well informed	5279	5251
Not very well informed	835	837
Not informed at all	288	285
DK/NA	109	109
**Total**	**8892**	**8892**

The question offers four categories, ranging from “very well informed” to “not informed at all”.

### 2.4 Training on safety and risks to health involved in the job

The presence or absence of training on the risks to the worker׳s health and safety posed by his/her job is the topic of question Q-49. It is asked as a Yes/No question, the results of which are presented in Table 6.

**Table 6:** Question Q-49 on the NSWC.

**Training on risks/safety**	No. of answers	
**Q-49**	Weighted	Unweighted
Yes	5085	5171
No	3700	3618
DK/NA	106	103
**Total**	**8892**	**8892**

### 2.5 Accidents reported in the previous two years

Variable that reflects if the worker reported having been in a work-related accident in the previous two years. This variable shows that 12 of the workers surveyed did not respond to this question, as a result of which these respondents were eliminated from the values for the remaining variables. The totals are shown in Table 7.

**Table 7:** Question Q-52 on the NSWC.

**Accident reported**	No. of answers	
**Q-52**	Weighted	Unweighted
Yes	670	686
No	8210	8194
DK/NA	12	12
**Total**	**8892**	**8892**

### 2.6 Visited doctor due to work-related health problem

In question Q-54, workers are asked about health problems. This question is in turn divided into three questions with independent answers. Our study only uses the data from the third sub-question (Q-54-C), which relates these health problems to the performance of the work activity and to the statement of having visited the doctor as a result. The survey data determine the specific ailment affecting the worker (15 options), as reflected in Table 8, but we attempted to quantify how many of them visited a doctor independently of the problem indicated, by considering the binary (yes/no) variable. Moreover, a positive answer was counted as a single instance even if the respondent visited the doctor for more than one problem at once.

**Table 8:** Question Q-54-C on the NSWC.

**Visited doctor for a health problem**	Weighted		Unweighted	
**Q-54-C**	Yes	No	Yes	no
Q54_C1	2476	6416	2481	6411
Q54_C2	3897	4995	3893	4999
Q54_C3	2063	6829	2078	6814
Q54_C4	1649	7243	1655	7237
Q54_C5	131	8761	134	8758
Q54_C6	286	8606	290	8602
Q54_C7	129	8763	130	8762
Q54_C8	214	8678	224	8668
Q54_C9	510	8382	514	8378
Q54_C10	182	8710	184	8708
Q54_C11	766	8126	761	8131
Q54_C12	577	8315	582	8310
Q54_C13	1254	7638	1254	7638
Q54_C14	262	8630	261	8631
Q54_C15	1469	7423	1470	7422

The data for this question are shown in Table 9.

**Table 9:** Summary Q-54-C.

**Visited doctor for a health problem**	Unweighted
**Summary Q-54-C**	No. of answers
Yes	5274
No	3618
**Total**	**8892**

### 2.7 Area of activity of the work center

Belonging to a specific production sector affects a worker׳s initial probability of contributing to the accident rate. This variable is defined in the survey by question Q-08, which applied the code in the 2009 National Classification of Economic Activities (CNAE) [13].

The question offers 87 activity categories considered in the 2009 CNAE. Since any process involving so many possibilities is ineffective, the data provided by the survey offer their own classification basis, with the answers grouped according to the four main areas of production:

• Agriculture.
• Industry.
• Construction.
• Services.

The results are shown in Table 10.

**Table 10:** Question Q-08 on the NSWC.

**Area of activity**	Weighted		Unweighted	
**Q-08 Grouped**	No. of answers	Percent	No. of answers	Percent
Agriculture	352	4.0%	457	5.1%
Industry	1222	13.7%	1448	16.3%
Construction	691	7.8%	599	6.7%
Services	6627	74.5%	6388	71.8%
**Total**	**8892**	**100.0**	**8892**	**100.0**
